# *In-situ* Simulation Use for Rapid Implementation and Process Improvement of COVID-19 Airway Management

**DOI:** 10.5811/westjem.2020.7.48159

**Published:** 2020-09-24

**Authors:** Brendan W. Munzer, Benjamin S. Bassin, William J. Peterson, Ryan V. Tucker, Jessica Doan, Carrie Harvey, Nana Sefa, Cindy H. Hsu

**Affiliations:** * Michigan Medicine, Department of Emergency Medicine, Ann Arbor, Michigan; † Michigan Center for Integrative Research in Critical Care, Ann Arbor, Michigan

## Abstract

**Introduction:**

The coronavirus disease 2019 (COVID-19) pandemic presents unique challenges to frontline healthcare workers. In order to safely care for patients new processes, such as a plan for the airway management of a patient with COVID-19, must be implemented and disseminated in a rapid fashion. The use of *in-situ* simulation has been used to assist in latent problem identification as part of a Plan-Do-Study-Act cycle. Additionally, simulation is an effective means for training teams to perform high-risk procedures before engaging in the actual procedure. This educational advance seeks to use and study *in-situ* simulation as a means to rapidly implement a process for airway management in patients with COVID-19.

**Methods:**

Using an airway algorithm developed by the authors, we designed an *in-situ* simulation scenario to train physicians, nurses, and respiratory therapists in best practices for airway management of patients with COVID-19. Physician participants were surveyed using a five-point Likert scale with regard to their comfort level with various aspects of the airway algorithm both before and after the simulation in a retrospective fashion. Additionally, we obtained feedback from all participants and used it to refine the airway algorithm.

**Results:**

Over a two-week period, 93 physicians participated in the simulation. We received 81 responses to the survey (87%), which showed that the average level of comfort with personal protective equipment procedures increased significantly from 2.94 (95% confidence interval, 2.71–3.17) to 4.36 (4.24–4.48), a difference of 1.42 (1.20–1.63, p < 0.001). There was a significant increase in average comfort level in understanding the physician role with scores increasing from 3.51 (3.26–3.77) to 4.55 (2.71–3.17), a difference of 1.04 (0.82–1.25, p < 0.001). There was also increased comfort in performing procedural tasks such as intubation, from 3.08 (2.80–3.35) to 4.38 (4.23–4.52) after the simulation, a difference of 1.30 points (1.06–1.54, p < 0.001). Feedback from the participants also led to refinement of the airway algorithm.

**Conclusion:**

We successfully implemented a new airway management guideline for patients with suspected COVID-19. *In-situ* simulation is an essential tool for both dissemination and onboarding, as well as process improvement, in the context of an epidemic or pandemic.

## INTRODUCTION

Epidemics and pandemics present numerous challenges to frontline healthcare workers. These providers must not only take care of patients during a period of uncertainty but must also ensure they protect themselves from exposure. The coronavirus disease 2019 (COVID-19) pandemic has led to the need for new management protocols to be created and implemented rapidly, including clinical guidelines related to the safety of healthcare workers.^1^ In the emergency department (ED), aerosol-generating procedures (AGP), such as endotracheal intubation of patients with presumed/confirmed COVID-19, represent the highest risk to healthcare providers due to the aerosolization of viral particles.^2^,^3^ These new guidelines must be quickly tested and disseminated in order to provide safe care.

The process of implementing a new management guideline can take significant time and buy-in from key stakeholders. New protocols typically develop through an iterative process, often in the form of a rapid Plan-Do-Study-Act (PDSA) cycle.^4^,^5^ Through a PDSA process, an educational, operational, or other need is identified and a process designed to fix it. After this initial implementation, feedback is obtained and studied. The initial process is then refined, restarting the cycle. In-situ simulation has previously been shown to be a powerful tool for identifying and correcting latent safety threats as well as process improvements in new hospital units and protocols.^6^–^8^ By using simulation within the space where that process occurs, new guidelines can be tested by those most affected, and comments can be fed back to revise the current workflow.^9^

Simulation is useful not only for process improvement but also to prepare teams for critical events. Prior work has shown that care teams have a better understanding of job responsibilities and improved communication during trauma activations after participating in an in-situ simulation.^10^ Surgeons use “simulation-based clinical rehearsals” to practice high-risk procedures prior to performing them on actual patients.^11^,^12^ Similarly, the use of “just-in-time” simulation training, which refers to the opportunity to practice a skill immediately prior to use in the clinical environment, has been shown to be effective in teaching skills and providing refreshers to avoid skill decay.^13^–^15^

The COVID-19 pandemic provides unique circumstances surrounding the implementation of new management guidelines and methods for teaching a large cohort of providers the skills necessary to deliver care in a safe manner. Due to state and federal executive orders prohibiting large gatherings, effectively leading to the cessation of typical in-person learning opportunities for providers, alternative methods for teaching are required.^16^,^17^ This brief innovation details our model for implementing an algorithm for managing the high-risk AGP in patients with presumed COVID-19 diagnosis and highlights our method for both rapidly refining our algorithm through a PDSA process and onboarding our providers to this new management protocol while following social distancing guidelines.

## METHODS

### Simulation Scenario

Our airway algorithm was developed by this authorship group (BSB and CHH) in coordination with hospital leadership and the Department of Anesthesiology (Supplemental File).^18^ To facilitate rapid PDSA cycling of this protocol and to onboard attending and resident physicians to new airway management guidelines, we developed an in-situ simulation scenario featuring a decompensating patient with COVID-19 requiring definitive airway management with intubation. The scenario was designed to fulfill the following primary objectives: 1) demonstrate and adhere to donning and doffing of personal protective equipment (PPE) for high-risk AGPs in suspected COVID-19 patients; 2) perform an AGP while maintaining precautions, including pre-brief, intubation, and post-intubation management; and 3) demonstrate closed-loop communication with an interprofessional team with PPE in place and ongoing infection control procedures. The scenario design process as well as the case itself are further detailed and available for use through the Association of American Medical Colleges iCollaborative.^19^ We developed and reviewed the scenario prior to implementation by educational leadership within the physician, nursing, and respiratory therapy groups.

In anticipation of a surge in critically ill patients requiring AGP, we conducted in-situ simulation sessions three times daily, prior to the start of clinical shifts. After one week, sessions were reduced to twice daily. These sessions occurred at the Michigan Medicine Adult Emergency Department, using a resuscitation room that was similar to rooms where patients would be intubated. Exact room was determined at the time of the session, based on room availabilities. Through announcements via email as well as during virtual departmental meetings, we invited all physicians, nurses, and respiratory therapists to participate in order to delineate roles and promote team communication. To comply with guidelines to minimize large gatherings, sessions were limited to the providers who were going to be working in the resuscitation area during the oncoming shift. Simulations were limited to six participants in their typical roles, reflecting the number of providers caring for a patient who requires an AGP in our protocol (two physician providers, two nurses inside the room, one respiratory therapist, and one additional nurse outside the room). If additional providers working that day showed up to the session, they were allowed to observe, following recommended social distancing guidelines. Initial sessions were taught by two authors (BWM and CHH). Additional faculty and residents were subsequently recruited on a volunteer basis to teach these sessions and were provided instruction on teaching methods and observed for a session, following a train-the-trainers framework. To minimize additional infectious risk, teachers were encouraged to sign up to teach sessions that were to occur immediately prior to their own shifts.

Prior to deployment of the simulation sessions, the airway management algorithm was provided to all providers through a link in an online repository Box (Redwood City, CA), although it was not mandatory for providers to review prior to attending the session.^20^ Simulation sessions focused on introduction of the concepts of appropriate donning of PPE; preparation and planning for intubation; the intubation procedure; post-intubation management; and appropriate doffing of PPE. Following a pre-brief that reviewed the airway management algorithm and demonstration of key elements, the participants engaged in the simulation scenario as a team, using a deliberate practice framework to correct errors in real time, noted by a critical action checklist. Due to national shortages of PPE, simulated equipment, such as Styrofoam masks replicating N95s, were used to practice donning and doffing techniques. Following the session, participants underwent debriefing that reinforced the critical actions.

### Airway Algorithm Refinement

**Figure 1:**
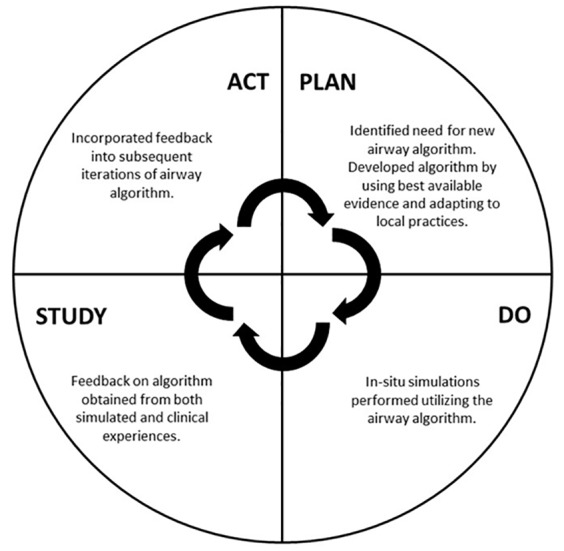
Plan Do Study Act cycle for refinement of the institutional airway algorithm for patients with suspected COVID-19.

The simulation sessions also informed the change process for the airway algorithm, following a PDSA cycle (Figure 1). After the initial implementation of the simulation, we sought feedback on the airway algorithm from participants and any observers present in real time regarding what worked well and how the algorithm could be improved. Additionally, providers were encouraged to email us with any additional feedback based upon their experiences in the clinical environment. This feedback was shared with the entire authorship group, who reviewed the information and used it to inform subsequent iterations of the airway algorithm. As new knowledge regarding best practices became available, this was also incorporated into new versions of the algorithm. We provided updated guidelines in Box for learners to review and refer to as needed.

Following participation in the simulation, the physician participants were asked to complete a retrospective pre/post survey using a five-point Likert scale (1 being extremely uncomfortable, 3 being neither comfortable nor uncomfortable, and 5 being extremely comfortable) regarding their comfort with aspects of the management of AGPs in COVID-19 patients before and after the simulation. Questions included physician level of comfort both before and after the simulation in the following domains: 1) PPE donning and doffing procedures; 2) understanding their role in AGPs; and 3) performing aerosol-generating procedural tasks such as intubation. We determined the mean and 95% confidence intervals (CI) of the survey results, and evaluated the pre- and post-simulation results using two-sided paired t-tests. All statistical computations were performed in SAS v9.2 (SAS Institute; Cary, NC). P value < 0.05 was considered statistically significant. We measured onboarding and reach through attendance at sessions by ED residents and faculty compared to those currently working in the department. Residents who were on off-service rotations and faculty who did not work shifts during the months of March and April were excluded. This study was exempted from institutional review board review by the University of Michigan Medical School Office of Research.

## RESULTS

Between March 16–April 1, 2020, 93 physicians completed the simulation training through a total of 37 simulation sessions. Of these physicians, 91.4% (85) were emergency physicians, while 8.6% (eight) were intensivists or anesthesiologists who attended sessions for the purpose of training their own departments in this algorithm. Of the ED providers, 45.9% (39) were residents or fellows and 54.1% (46) were attending physicians. This represented 86.7% (39 of 45) residents and 83.6% (46 of 55) of faculty who worked shifts in the ED during this time.

We received 81 responses from the 93 participants (87% response rate). Thirty (37%) of the providers had participated in an AGP on a suspected or confirmed COVID-19 patient prior to participating in the simulation. The average level of comfort with PPE procedures increased significantly from 2.94 (95% CI, 2.71–3.17) to 4.36 (4.24–4.48), a difference of 1.42 (1.20–1.63, p < 0.001). The providers again showed a significant increase in average comfort level in understanding their role with scores increasing from 3.51 (3.26–3.77) to 4.55 (2.71–3.17), a difference of 1.04 (0.82–1.25, p < 0.001). In addition, providers showed significantly increased comfort in performing procedural tasks such as intubation. Their comfort level increased from 3.08 (2.80–3.35) before to 4.38 (4.23–4.52) after the simulation, a difference of 1.30 points (1.06–1.54, p < 0.001) (Table 1). There was no significant difference in the above scores between the providers who had participated in AGPs in suspected or confirmed COVID-19 patients, and those who had not (p-values 0.33, 0.41, and 0.45, respectively) (Table 2).

During the study period, we created a total of 12 versions of the COVID-19 airway algorithm. Changes occurred in response to several avenues of feedback, as described in the methods. Ever-changing consensus recommendations related to airway management in a new disease required maximal flexibility and adaptability. New knowledge regarding best practices were adapted as they became available. Additionally, all participants were offered the opportunity to provide suggestions for change after participating in the simulation. The airway algorithm was also used by many participants on clinical shift immediately following completion of simulation, leading to the discovery of areas needing refinement. To track modifications to the algorithm we created a descriptive “Change Log,” which is represented in Table 3.

## DISCUSSION

The COVID-19 pandemic presents a unique scenario in which new management guidelines must be implemented in a rapid manner to provide healthcare workers the tools necessary to safely perform patient care. The use of *in-situ* simulation allowed for the simultaneous training and refinement of our airway algorithm. Provider comfort in multiple domains improved significantly following the simulation, independent of whether the providers had participated in an AGP in a suspected or confirmed COVID-19 patient prior to participating in the simulation training. These domains included PPE donning and doffing; knowing one’s role in AGPs; and performing AGPs such as intubations.

During this time, multiple changes took place to the airway algorithm and several themes were noted throughout the revision process. The importance of proper PPE donning/doffing was recognized during initial algorithm development; however, defining the order and type of PPE were continually assessed and modified. Communication barriers were uncovered including need for two-way communication devices. Layout of airway equipment was redesigned from an initial airway bag to a preset airway table and, finally, to separate, modular airway packs.

This study highlights the importance of *in-situ* simulation training, particularly its impact on provider confidence with high-risk AGPs such as intubation, as well as team roles and PPE donning and doffing. Additionally, it shows that an airway algorithm can be developed and refined in real time based on user feedback and rapidly disseminated to ED providers. Future work will look at the impact of the training on provider outcomes such as adherence to PPE donning and doffing standards, as well as patient outcomes such as success of airway interventions. Additionally, further analysis of data will look at any differences between the original trainers and the secondary teachers to evaluate the quality and consistency of the sessions.

## LIMITATIONS

The need to follow social distancing guidelines presented a significant limitation in the number of providers we were able to train at one time. In the setting of a pandemic, access to supplies and equipment was unpredictable. One limitation was the need to adapt to what was available and in stock. Although other hospitals may not be able to reproduce exactly our airway algorithm, the process for implementation is generalizable. Additionally, the recommendations we provided to our learners were best practice recommendations as there was limited evidence supporting a definitive management algorithm in the context of the COVID-19 pandemic.

We did not ask the participants to review the airway algorithm prior to attending the session, although some may have done so. Given that we did not collect data on whether or not participants were familiar with the algorithm prior to the simulation, our results may underestimate the utility of the simulation training. Additionally, as this was a retrospective survey, there is the possibility of recall bias as it relates to participant comfort with the procedure. Although future work will assess whether or not different instructors were more effective teachers, it was not a part of this study and therefore is a potential limitation in understanding the dissemination of content.

## CONCLUSION

We successfully implemented a new airway management guideline for patients with suspected COVID-19. The use of *in-situ* simulation helped providers learn these new guidelines and become familiar with new equipment and protocols over a short time period. Additionally, the feedback obtained through the simulation was useful in refining our algorithm. In-situ simulation is an essential tool for both rapid dissemination and onboarding, as well as process improvement in the context of an epidemic or pandemic.

## Supplementary Information



## Figures and Tables

**Table 1:** Survey questions and results with means and pre/post intervention differences.

Question	Pre-Intervention Mean (95% CI)	Post-intervention Mean (95% CI)	Difference (95% CI)	P-value
How comfortable did you feel in appropriately donning and doffing PPE in an AGP in a suspected COVID-19 patient?	2.94 (2.71 – 3.17)	4.36 (4.24 – 4.48)	1.42 (1.20–1.63)	<0.001
How comfortable did you feel in knowing your role in the management of an AGP in a suspected COVID-19 patient?	3.51 (3.26 – 3.77)	4.55 (4.42 – 4.68)	1.04 (0.82–1.25)	<0.001
How comfortable did you feel in performing your responsibilities (intubating, giving medications, transitioning patient to vent, etc) without violating PPE precautions during the management of an AGP in a suspected COVID-19 patient?	3.08 (2.80 – 3.35)	4.38 (4.23 – 4.52)	1.3 (1.06–1.54)	<0.001

*AGP*, aerosol-generating procedure; *PPE*, personal protective equipment; *CI*, confidence interval.

**Table 2:** Provider comfort with the following after simulation based on whether had performed procedure in patient.

	Donning and doffing PPE mean (95% CI)	Difference (95% CI)	P-value
Had performed procedure prior (n = 30)	4.43 (4.25 – 4.62)	0.12 (−0.16 – 0.44)	0.33
Had not performed procedure prior (n = 51)	4.31 (4.15 – 4.48)		

	Knowing role in management of AGP mean (95% CI)	Difference (95% CI)	P-value

Had performed procedure prior (n = 30)	4.62 (4.41 – 4.83)	0.11 (−0.13 – 0.40)	0.42
Had not performed procedure prior (n = 51)	4.51 (4.34 – 4.68)		

	Performing AGP and maintaining PPE mean (95% CI)	Difference (95% CI)	P-value

Had performed procedure prior (n = 30)	4.45 (4.21 – 4.69)	0.12 (−0.12 – 0.37)	0.45
Had not performed procedure prior (n = 51)	4.33 (4.14 – 4.53)		

*AGP*, aerosol-generating procedure; *PPE*, personal protective equipment; *CI*, confidence interval.

**Table 3:** COVID-19 airway algorithm change log.

	Preparation	Pre-brief	Procedure	Post-procedure	Equipment
1	Move patient to negative pressure room Identify the team: 2 airway operators, 2 nurses, 1 respiratory tech, 1 runner, 1 PPE monitor Check equipment in airway bag Don PPE	Discuss plan, including pre-oxygenation, RSI medications and post-intubation sedation plan	Avoid providing BVM oxygenation unless life threatening hypoxemia Intubate with RSI and VL Use an iGel with a viral filter if need for re-oxygenation Avoid ventilation until ETT cuff inflation	Confirm ETT placement with ETCO^2^ Transfer to ventilator by clamping ETT to connect to circuit Discard equipment and wipe down Glidescope Doff PPE with the assistance of the PPE monitor	Glidescope BVM with ETCO^2^ adapter and viral filter for preoxygenation and rescue breathing Airway bag containing airway equipment, nursing supplies, and respiratory therapist supplies
2	Updated PPE guidelines to remove shoe covers due to concern for self-contamination and to include goggles Identified specific Glidescope for AGP	Expanded RSI medications and clarified recommended doses		Clarified doffing procedure to specify hand hygiene between each step	Added two way communication device between team in room and outside Changed airway bag to preset airway table
3	Clarified that post-sedation medications should be primed prior to entering room		Specified that heated high flow nasal cannula should be turned off prior to intubation Emphasized that cuff should be inflated prior to positive pressure ventilation	Clarified appropriate doffing order	
4					Updated airway table to include labels for ease of use and restocking Updated ventilator circuit to remove extraneous viral filter
5	Clarified order of donning PPE			Updated guidelines to wipe down unopened equipment for reuse	
6	Updated order of donning PPE				
7					Changed tube clamps to plastic due to metal clamps cracking ETT
8			Adjusted pre-oxygenation method with BVM to accommodate lack of bidirectional flow of oxygen.		Removed disposable stethoscope from airway table Added cover to table to signify that it was ready for use Added sterile cover to two way communication device for ease of cleaning
9			Clarified the process for attaching the BVM to the ETT	Added clarification on process for cleaning equipment and order for doffing PPE	
10	Face shield added to donning procedure	Expanded recommendations for post-intubation sedation	Changed pre-oxygenation option from 6L nasal cannula to 15L green nasal cannula Clarified order and speed of RSI medications		Changed airway table to modular airway packs
11	Clarified role responsibilities in obtaining airway packs Removed role stickers from bags Added additional changing of gloves during donning of PPE to accommodate reuse of N95 mask	Added code starter pack for medications			
12		Clarified medication plan for hemodynamic optimization		Clarified procedure for cleaning equipment in and out of room as well as restocking of airway packs	Added rescue cart available outside of room

*AGP*, aerosol-generating procedure; *BVM*, bag valve mask; *ETCO*^2^, end tidal carbon dioxide; *ETT*, endotracheal tube; *PPE*, personal protective equipment; *RSI*, rapid sequence intubation; *VL*, video laryngoscopy.
