# Supplementary material for: In-situ Simulation Use for Rapid Implementation and Process Improvement of COVID-19 Airway Management
Source: West J Emerg Med. 2020 Sep 24;21(6):99–106. doi: 10.5811/westjem.2020.7.48159 (PMC7673893; doi:10.5811/westjem.2020.7.48159)
Supplement: Supplementary file 1 [file wjem-21-99-s001.pdf]

Michigan Medicine Department of Emergency Medicine

COVID-19 AIRWAY MANAGEMENT ALGORITHM

1. PREPARATION

LOCATION AND TIMING

Negative Pressure Room if possible

Consider early intubation given time needed for preparation

If potential difficult airway notify Anesthesia early given time for response and PPE prep time.

ASSEMBLE TEAM

In-Room	Out-of-Room
2 Experienced airway operators	1 Runner/PPE Monitor (in full PPE)
2 Resus/EC3 nurses	EC3 Team Lead to read off this algorithm
1 RT	

Minimize number of healthcare providers needed to complete procedure safely while maximizing protection

PPE

HAND HYGIENE for at least 20 seconds

In-Room personnel, Runner/PPE Monitor must **DON**:

Inner gloves
↓
Impermeable gown w/ thumbs through thumb holes
↓
Outer gloves
↓
N95 or PAPR
↓
Doff outer gloves
↓
Hand hygiene
↓
Don new outer gloves
↓
Face shield
↓
OR cap

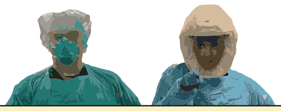

PPE Monitor

Supervise all donning & doffing of PPE to ensure no cross contamination

Last Edited: April 3, 2020

2. PRE-CHECK & PRE-BRIEF

EQUIPMENT CHECK

- COVID glidescope is charged and working
- Two-way communication device is active
- Secure a procedure table
- Airway cart is outside of the room
- **Airway Team**: Select **Plan A Airway Pack**. Obtain **Plan B Airway Pack**, **Nursing**, and **RT packs**. Add Plan C Airway Pack if anticipating difficult airway

PRE-OXYGENATION PLAN

Determine the optimal pre-oxygenation strategy. Options include:

15L/min O2 Green NC with surgical mask on patient

OR

BVM + PEEP valve with 2-hand tight mask seal, viral filter, **6L/min O2 connected to ETCO2 adapter,** and 15L/min inlet O2. Use PEEP as needed. **DO NOT BAG**.

OR

HFNC up to 50L/min using the Drager with surgical mask on patient

INTUBATION PLAN

Plan A: RSI with VL

Provider with best chance for first past success should intubate

Plan B: Rescue Oxygenation

i-gel with viral filter between i-gel and BVM. Bag with 15L/min inlet O2 and PEEP needed for re-oxygenation. If fails, bag w/o i-gel but must ensure 2-hand tight seal with in-line viral filter.
Use OPA/NPA as needed

Plan C: Front of Neck Access

Scalpel, bougie, 6-0 ETT

MEDICATION PLAN

In-Room: RSI Use Ketamine (0.5 - 1 mg/kg) or Etomidate (0.3 mg/kg) and high-dose Rocuronium (1.2-1.6 mg/kg) or Succinylcholine (1.5-2.0 mg/kg) to suppress gag/cough and optimize intubating conditions

In-Room: Sedation - Pre-prime Propofol or Midazolam and Fentanyl gtt. Bring pump into the room

Out-of-Room: Hemodynamic optimization - Norepinephrine and epinephrine gtt

Out-of-Room: Code Starter Pack -Epinephrine, sodium bicarbonate, phenylephrine, calcium chloride

3. PROCEDURE

ORGANIZE

- Personnel, Airway Packs, table, medications, and glidescope into the room
- Door closed
- Set up viral filter and ETCO2 in-line on BVM and ventilator circuit (see photos)
- Set up closed suctioning system (Yankauer) with tight seal on canister
- BP cuff set for q3 min and opposite arm from pulse ox

OPTIMIZE

- Correct hypotension, hypoxemia, and acidosis
- Pre-oxygenate using the pre-determined strategy
- Use wedge as needed to optimize airway anatomy with ear-to-sternal notch position
- If patient is agitated, consider small dose of IV ketamine (10-30mg)

INDUCTION AND INTUBATE

- **PERFORM TIME OUT**
- Administer RSI meds (**induction FIRST** then paralytic) as **RAPID, sequential pushes**, then wait 1 min. Do not bag during apneic period if possible.
- For life-threatening hypoxia: Bag with 2-hand tight seal or insert i-gel for rescue oxygenation prior to intubation attempt
- Turn off HFNC if applicable then take off surgical mask.
- **Intubate**
- Inflate ETT cuff FIRST → Clamp ETT as stylet removed → attach BVM → Unclamp ETT → Bag

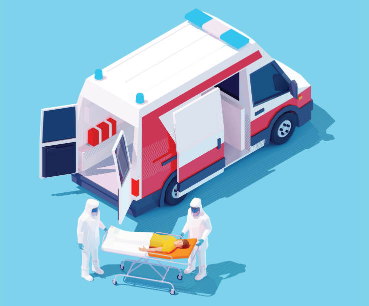

MICHIGAN MEDICINE
UNIVERSITY OF MICHIGAN

4. POST CHECKS

TUBE SAFE?

- **Confirm ETCO2 waveform and secure ETT**
- **Transfer to vent:**
  Clamp ETT → remove BVM → connect ETT to vent → unclamp ETT
  Ensure EtCO2 monitor is in-line.
- **Planned disconnections:**
  Always put ventilator in Standby Mode and clamp ETT prior to disconnecting

BRAIN / HEART SAFE?

- Start analgosedation
- Send ABG/VBG, correct acidosis
- HOB 30 degrees

LUNGS SAFE?

- TV < 6-8 mL/kg IBW
- Pplat < 30cm H2O
- Adequate expiratory time/autoPEEP
- Insert OG tube

STAFF SAFE?

In Room

- Place glidescope blade and any soiled equipment in red bag. Seal and leave in room
- Doff outer gloves → Hand hygiene → Don new outer gloves.
- Wipe glidescope, table, and unused Airway Packs with OxiVir. Put unused equipment into the "dirty" bin.
- Push glidescope and table out of room w/ wipe-in-hand
- Doff gown and outer gloves → Exit room

Out-of-Room:

- Doff inner gloves → Hand hygiene → Don clean gloves → Doff cap or PAPR → Hand hygiene → Doff face shield → Hand hygiene → Doff N95 → Doff gloves → Hand hygiene → Wash face w/ soap/water
- Glidescope, table, and unused Airway Packs wiped down again by Runner/PPE monitor

5. DEBRIEF
